# Pharmacologically altered androgens, brain activation and response inhibition in a stop-signal task in male heavy drinkers

**DOI:** 10.3389/fnsys.2026.1907416

**Published:** 2026-10-01

**Authors:** Marlene Kundlacz, Rafat Boroumand-Jazi, Sabine Hoffmann, Christiane Mühle, Iris Reinhard, Matthias Reichl, Leonard Wenger, Haoye Tan, Patrick Bach, Sabine Vollstädt-Klein, Georg W. Alpers, Falk Kiefer, Bernd Lenz, Sarah Gerhardt

**Affiliations:** 1 Department of Addictive Behavior and Addiction Medicine, Central Institute of Mental Health (CIMH), Medical Faculty Mannheim, Heidelberg University, Mannheim, Germany; 2 Otto Selz Institute and Department of Psychology, School of Social Sciences, University of Mannheim, Mannheim, Germany; 3 Department of Psychiatry and Psychotherapy, Universitätsklinikum Erlangen and Friedrich- Alexander-Universität Erlangen-Nürnberg (FAU), Erlangen, Germany; 4 Core Facility Biostatistics, Central Institute of Mental Health (CIMH), Medical Faculty Mannheim, Heidelberg University, Mannheim, Germany; 5 Deutsches Zentrum für Psychische Gesundheit (German Center for Mental Health), Partner Site Mannheim-Heidelberg-Ulm, Mannheim, Germany; 6 Mannheim Centre for Translational Neurosciences (MCTN), Medical Faculty Mannheim, Heidelberg University, Mannheim, Germany; 7 Feuerlein Center on Translational Addiction Medicine (FCTS), Heidelberg University, Mannheim, Germany

**Keywords:** alcohol, DHT, inhibitory control, neuroimaging, testosterone

## Abstract

Heavy episodic drinking (HED) is highly prevalent in men and a major risk factor for Alcohol Use Disorder (AUD). Previous studies indicated an influence of testosterone (T) on AUD, and dihydrotestosterone (DHT) was linked to alcohol consumption, impulsivity, and response inhibition. As the role of androgens in AUD and the modulation thereof is not fully understood, this study investigated the effect of a pharmacological reduction in DHT by inhibition of the 5-alpha reductase II with finasteride on brain activation and response inhibition during a stop signal task. Fifty males with HED participated in a randomized, placebo-controlled, double blind, cross-over, functional magnetic resonance imaging (fMRI) study. Participants received either 5 mg finasteride or placebo, resulting in a 9.6% reduction of DHT levels. Full factorial fMRI models and linear mixed models were used to estimate whole-brain effects of DHT and testosterone following finasteride and placebo on neural correlates of behavioral control (*N* = 47 analyzable participants). While no medication effects were observed on behavioral or neural levels, lower levels of DHT and higher levels of testosterone were related to stronger neural activation in temporal regions during response inhibition for successful or unsuccessful stop-trails, respectively. However, no significant influence of hormone levels on behavioral data of the stop signal task was observed. Although the precise mechanisms underlying the effects of pharmacologically altered DHT levels on neural correlates of response inhibition remain unresolved, both DHT and testosterone appear to be specifically associated with neural activation during response inhibition. These preliminary findings may provide a rational for future studies investigating pharmaceutical modulation of androgens as a potential therapeutic approach for problem drinking, and potentially AUD.

**Clinical trial registration:**

https://drks.de/search/de/trial/DRKS00020569, identifier DRKS00020569.

## 1 Introduction

Alcohol consumption is considered a significant health issue worldwide, and sex differences are recognized regarding prevalence of alcohol consumption and alcohol use disorder (AUD). Even though the gap between men and women is narrowing, 56.5% of men in Europe above the age of 15 years reported heavy drinking episodes [HED, defined as consuming at least 60 g of pure alcohol on one drinking occasion at least once per month, (World Health Organization, 2018)], as compared to 24.5% of the women. HED constitutes a major risk factor of AUD, as about 42% of individuals with HED develop AUD (Linden-Carmichael et al., 2017).

A multifactorial etiology of the disorder is assumed, and several studies implicated an important contribution of sex hormones. A systematic review observed that both, higher blood or salivary levels of testosterone, are generally related to higher levels of alcohol consumption (Erol et al., 2019). Likewise, higher total blood testosterone levels are associated with increased risk of alcohol consumption (Erol et al., 2019). Male patients with AUD further have significantly higher levels of testosterone than healthy controls (Heberlein et al., 2016). Dihydrotestosterone (DHT), the activated derivative of testosterone in the human body, is up to six times more potent than testosterone and binds more strongly to the androgen receptor (Stárka et al., 2009). An increase in DHT levels during acute withdrawal treatment has been found to predict a worse 12-months outcome in male and female patients with AUD in terms of more and earlier alcohol-related hospital readmissions (Lenz et al., 2017).

Nave et al. (2017) showed that higher testosterone levels also correlate with lower impulse control and impaired decision-making in healthy males. Impulsivity, as the overarching construct of impulse control, and response inhibition as an endophenotype thereof (Bari and Robbins, 2013; King et al., 2014), have been shown to play an important role in the initiation and continuation of alcohol consumption and the development of AUD, for example by influencing the ability to inhibit unwanted behaviors (Lejuez et al., 2010; Whelan et al., 2012). The most widely used task to assess response inhibition, as well as its neural correlates, is the stop signal task (SST) (Logan et al., 2014). Functional magnetic resonance imaging (fMRI) studies using the SST observed that patients with AUD show less activation of regions responsible for impulse control, e.g., caudate (Bednarski et al., 2012); opercular part of the inferior frontal gyrus (Gan et al., 2014); subgenual anterior cingulate cortex (Li et al., 2009); anterior insula (Weafer et al., 2021). Although androgens have been implicated in alcohol consumption and AUD, the neural mechanisms through which they may influence response inhibition remain poorly understood. Previous studies have demonstrated a negative impact of alcohol consumption on response inhibition and its neural correlates (Bednarski et al., 2012; Gan et al., 2014; Li et al., 2009; Weafer et al., 2021). Likewise, the relationships of higher testosterone levels and less inhibitory control as well as higher alcohol consumption are acknowledged (Lejuez et al., 2010; Nave et al., 2017). This leads to the assumption that lower levels of DHT (the potent derivative of testosterone) might positively influence behavioral control in individuals, especially males, with HED via underlying neural mechanisms, such as stronger activation of the left inferior parietal lobe in males compared to females (corresponding to stronger performance in mental rotation and spatial tasks) (Celec et al., 2015).

The present study explores the effects of lowered DHT levels induced by pharmacological inhibition of 5-alpha reductase II by finasteride on neural and behavioral correlates of response inhibition in males with HED. A recent study reported reduced alcohol intake in heavy drinking males under treatment with dutasteride, another 5-alpha reductase inhibitor (Covault et al., 2024), supporting the need for explaining underlying processes.

We thus hypothesize that reduced DHT levels associate with increased neural activation in brain regions related to behavioral control during response inhibition. Although the existing literature is sparse, available evidence points toward fronto-striatal circuitry as a plausible neural substrate. In exploratory analyses, we further analyze the influence of testosterone levels as a predictor as well as behavioral data during response inhibition as outcome. The study only included males, who exhibit higher absolute levels of testosterone and a more consistent relationship between AUD and testosterone (Erol et al., 2019), as well as AUD and DHT (Lenz et al., 2017), as compared to females, therefore having a clearer impact of finasteride on circulating testosterone and DHT.

## 2 Materials and methods

### 2.1 Study design and participants

This randomized, placebo-controlled, double-blind, crossover finasteride challenge experiment was part of the TRR 265 A08 project funded by the German Research Foundation (Heinz et al., 2020). Recruitment of the preregistered study (DRKS00020569) was conducted between February 2022 (first study visit) and February 2023 (last study visit). Ethics approval was obtained from the local ethics committee II of Heidelberg University, Germany (approval number 2021-654). For the recruitment of participants, a databank of participants from the TRR265 was used, and an advertisement was sent via e-Mail to students at the University of Mannheim.

*N* = 50 males aged between 18 and 65 years were included in the study and randomized according to protocol, if they (1) had a body mass index between 18.5 and 30 kg/m^2^, (2) were able to provide written informed consent after having received detailed information, (3) fulfilled the criteria for heavy drinking episodes according to the World Health Organization (i.e., a minimum of 60 g of pure alcohol on a single occasion at least once per month), (4) were able to undergo fMRI measurements, and (5) met the requirements for the intake of a single dose of 5 mg of finasteride. For additional exclusion criteria, see Supplementary Material 1.

Participants were invited to four study visits (V1 – V4, see Figure 1). At V1, written informed consent was obtained, inclusion and exclusion criteria were checked, and participant information (sociodemographic information, substance use) were assessed. Participants were then randomized either to the finasteride – placebo or the placebo – finasteride sequence group. No demographic factors were included in the randomization procedure. V1 and V2 took place on two subsequent days (i.e., 12 to 36 h in between, on average 21 h in our sample). We scheduled V3 to take place after a washout phase of minimum 21 to maximum 42 days. The interval between V3 and V4 corresponded to that between V1 and V2. Whenever possible, visits were scheduled at the same time of day (e.g., V1 at 5:00 p.m. and V2 at 10:00 a.m. the following day; V3 at 5:00 p.m. 21 days later and V4 at 10:00 a.m. the following day). Participants received either placebo or 5 mg of finasteride at V1 and V3. At V2 and V4, fMRI measurements were conducted (fMRI measurement at V2 = T1, at V4 = T2). Blood samples were drawn at all four visits to assess DHT und testosterone hormone levels. After the completion of the study of the last participant, data was preprocessed prior to obtaining the randomization list to unblind the data.

**FIGURE 1:**
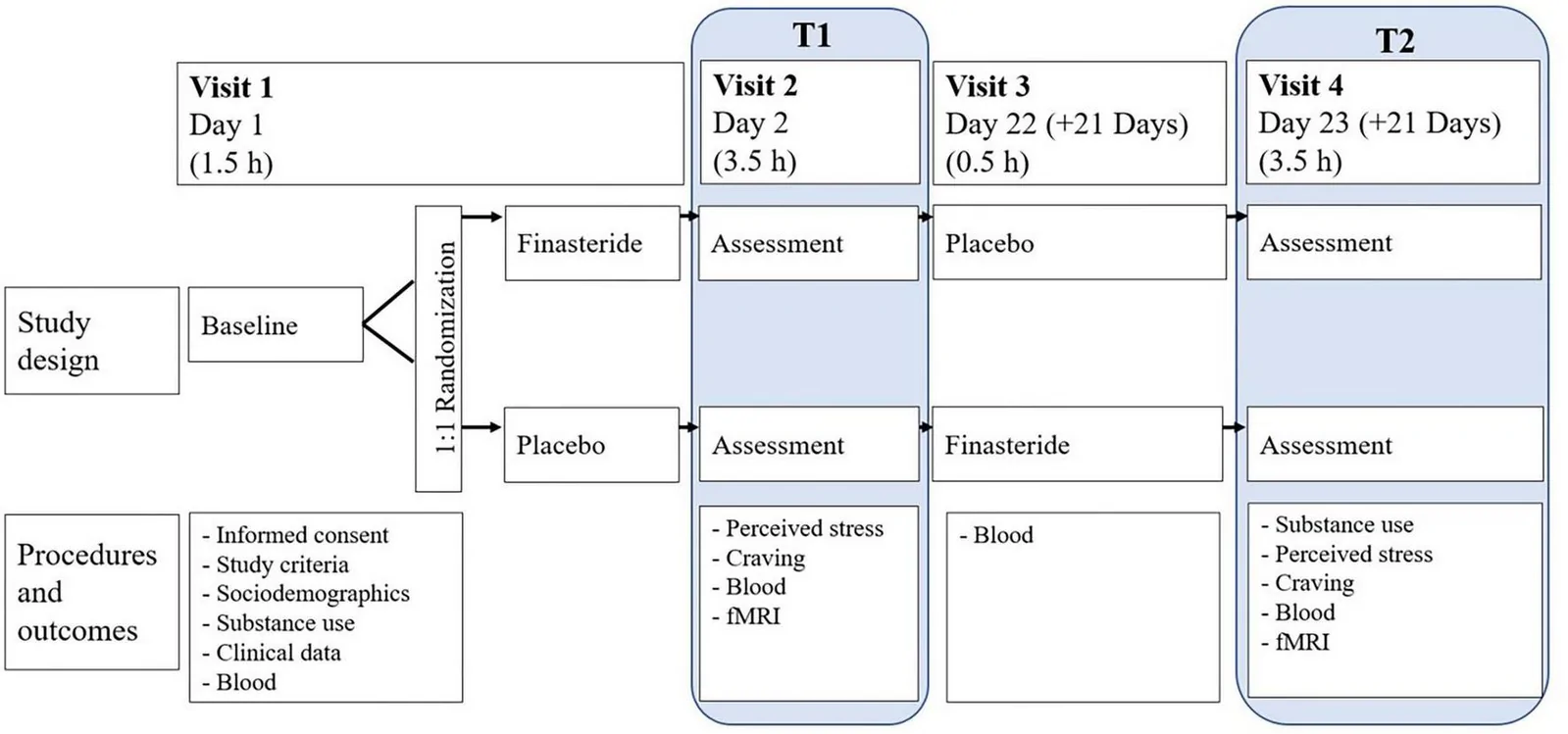
Schematic display of the study design. Visit 2 and 3 were scheduled with a washout phase of 21–42 days in between (M 25.8, SD 6.7 days).

### 2.2 Psychometric measurements

Psychometric measures included the Alcohol Use Disorder Identification Test at V1 and V3 [AUDIT; (Rist et al., 2003); retest reliability 0.86 (Dybek et al., 2006)] and the Barratt Impulsiveness Scale at V1 [BIS; (Meule et al., 2011); good intrascale reliability of 0.82; (Spinella, 2007)].

### 2.3 Medication

At V1 and V3, 5 mg of finasteride (Gisleskog et al., 1998) or placebo were administered. By mainly inhibiting the 5-alpha reductase II enzyme, which is necessary for the transformation of testosterone into its more potent derivative DHT, administration of finasteride has been suggested to decrease serum DHT levels by approximately 80% within approximately 1–2 days for up to 1–2 weeks (Gisleskog et al., 1998; Pope, 2023). A systematic review on finasteride did not observe changes in testosterone levels after the intake of finasteride, while DHT levels were significantly decreased in males and females (Lee et al., 2018). Finasteride can remain functionally active after single administration for up to 21 days (Gisleskog et al., 1998).

### 2.4 Stop signal task

The SST (Gan et al., 2014) was administered during an fMRI examination to assess response inhibition. During the task, participants responded to arrows pointing right or left by pressing the corresponding key on a remote control in their hand with their respective thumb. In 20% of trials, a “stop” signal in form of an arrow pointing upward appeared shortly after the arrow pointing right or left, requiring the participant to inhibit an initiated reaction of pressing a key (Logan, 1994). In total, the SST lasted 10:39 min and included 320 “go” and 80 “stop” trials (see Supplementary Material 6 for more details about the task).

### 2.5 Functional MRI acquisition

While performing the SST, 725 brain scans were acquired in a 3T whole-body tomograph at the Central Institute of Mental Health in Mannheim (MAGNETOM 3.0T XR Numaris, Siemens Medical Systems, Erlangen, Germany) using T2*-weighted multi-band echo-planar imaging (mb-EPI) sequences [repetition time (TR) = 869 ms, echo time (TE) = 38 ms, flip angle = 58°, 60 slices, slice thickness 2.4 mm, voxel dimensions 2.4 × 2.4 × 2.4 mm^3^, no inter-slice gap, field of view (FOV) = 210 × 210 mm^2^, matrix size 88 × 88 in-plane resolution, acquisition orientation T > C with 20° clockwise to anterior commissure – posterior commissure (AC-PC) line, interleaved slice order, acceleration factor slice = 6, flip angle = 58°, bandwidth = 1832 Hz/Px, prescan normalize, weak raw data filter, LeakBlock kernel, fat sat].

### 2.6 Blood sampling and hormonal analysis

To assess levels of DHT and testosterone at each study visit, venous blood samples were collected and concentrations were quantified in serum aliquots stored at −80 °C. Androgens were determined using a competitive enzyme-linked immunosorbent assay (ELISA) specified as follows [with indicated serum volumes in duplicates, standard ranges and achieved intra- and inter-assay coefficients of variation (cv)]: 5a-Dihydrotestosterone ELISA (DB52021; IBL International GmbH, Hamburg, Germany; 50 μl, standard 6–2500 pg/ml, 1.5%, 2.2%) and Testosterone ELISA (DRG-EIA-1559; DRG Instruments GmbH, Marburg, Germany; 25 μl, standard 0, 1–16 ng/ml, 1.9%, 3.4%).

### 2.7 Statistical analyses

A-priori sample size estimation was based on a previous publication examining inhibitory control in a placebo-controlled cross-over design using the SST in *N* = 50 alcohol consuming individuals (Gan et al., 2014) and previous studies with moderate to large treatment effect size of *d* = 0.71 and a power of 80% examining pharmacological effects in individuals with AUD [alpha = 0.05 (Hermann et al., 2006; Vollstädt-Klein et al., 2019a)]. Thus, expecting a moderate effect of pharmacologically altered androgens on neural correlates of response inhibition, we planned to include *N* = 50 participants. SPSS (Statistics for Windows, Version 29.0. IBM Corp., Armonk, NY, USA) was used to perform descriptive and statistical analyses. Linear mixed models analyzed behavioral data within the cross-over experimental study design. These analyses accounted for the nested structure of the data, as data from two measurement time points, T1 and T2, were available per participant. Separate mixed models were computed for the dependent variables stop signal delay (SSD), go reaction time (reaction time following a go-signal; GoRT), stop signal reaction time (SSRT, calculated with mean GoRT). We first analyzed models including only hormone level (DHT or testosterone after intake of medication or placebo) and age along with the dependent variables. Then, the factors time point (V2 and V4), medication (finasteride or placebo), and sequence (finasteride first or placebo first) were added to the linear mixed models.

### 2.8 fMRI pre-processing and first-level fMRI analyses

Functional magnetic resonance imaging data was preprocessed in SPM12 (Statistical Parametrical Mapping; Wellcome Center for Human Neuroimaging, at University College, London, UK) using a standardized pipeline including slice timing, spatial realignment, normalization (template: SPM12 tissue probability map according to the Montreal Neurological Institute), and spatial smoothing (Gaussian kernel of 8 mm full width at half maximum). Quality checks were performed and individuals with excessive head movement (>3 mm/3°) or other artifacts were excluded from further analyses (see Supplementary Material 2). On the first level (single subject), the obtained and pre-processed data were analyzed by modeling the three contrasts: “stop success,” “stop error” both contrasted against the implicit baseline of successful “go” trials, and “stop success vs. stop error.” This involved examining neural reactivity during successful and unsuccessful response inhibition, as well as the difference in reactivity between successful and unsuccessful inhibition. To this end, convolution with the canonical hemodynamic response function was performed, a high-pass filter (with a cutoff at 128 s) was applied, and a general linear model (GLM) was calculated on a voxel-by-voxel basis, including the task condition and six motion regressors.

### 2.9 fMRI whole brain analyses

Prior to the hypothesis-driven analyses, a whole brain one-sample *t*-test using data from the placebo intervention was conducted in SPM12 to confirm the successful implementation of the SST paradigm (contrasts “stop success,” “stop error,” and “stop success vs. stop error”). The time point of measurement (T1/T2 referring to V2/V4) was entered as covariate.

To test our hypothesis and to account for the cross-over design of the study, flexible factorial models were then calculated in SPM12 separately for each of the three contrast. Subject, time point (V2 and V4), medication (finasteride or placebo), and sequence (finasteride first or placebo first) were included as factors. Individual DHT or testosterone levels were included as covariates of interest. For exact contrast specification, see Supplementary Material 3.

Age was added as a covariate to all models, as brain activation is known to significantly differ depending on age in the context of response inhibition (Grady et al., 2006). To account for multiple testing, the analyses were corrected combing a voxel-wise-threshold of *p* < 0.001 with a cluster-extend-threshold determined with random field theory in SPM12 corresponding to a family-wise error (FWE)-rate of pFWE < 0.05.

## 3 Results

### 3.1 Sample characteristics

Eighty-five eligible participants were invited to participate in the study and a total of 50 participants were randomized to either the finasteride – placebo or the placebo – finasteride sequence following a randomization list of fifty group allocations. fMRI data was available from 47 participants (see CONSORT flow diagram, Supplementary Material 2 for more details regarding allocation and exclusion), with *N* = 32 participants having two analyzable fMRI data sets, and *N* = 15 only one. No severe adverse events were reported following intake of finasteride or placebo.

Participants were on average 33.5 years old (SD = 13.5). They exhibited a mean AUDIT sum score of 11.3 (SD = 5.0) indicating a hazardous and harmful consumption of alcohol. BIS scores (*M* = 30.1, SD = 5.4) were similar to those of a normative sample (*M* = 30.0, SD = 6.1). For additional sociodemographic data, see Table 1. No significant group differences regarding the medication sequence (finasteride or placebo first) were observed at baseline (see Supplementary Material 4, Supplementary Table 2). DHT levels were significantly reduced by 9.6% as compared to placebo [from 353 to 318 pg/mL on average; *F*(1, 42.02) = 12.50, *p* = 0.001]. No significant change in testosterone levels was observed [*F*(1, 42.24) = 1.05, *p* = 0.311)].

**TABLE 1:** Sample characteristics at V1.

	*M*	SD	% of sample	*N*
Age	33.5	13.5	70	33
High school diploma (“Abitur”)	11.3	5.0		
Employment in the last 3 months			94	44
Single/divorced/living apart: married/in relationship			70: 30	35: 14
Having children (yes)			34	16
Smokers			36	17
AUDIT (sum score)				
BIS (sum score)	30.1	5.4		

*M*, mean. *N*, number. SD, standard deviation. AUDIT, Alcohol Use Identification Test. BIS, Barratt Impulsiveness Scale. Heavy drinking days were defined as consuming at least 60 g of alcohol per drinking occasion.

### 3.2 fMRI results

The stop-signal paradigm was successfully applied, as demonstrated by the typical neural activation patters in the placebo only analyses, see Supplementary Table 3. Also, percentage of stop success rate varied between 40% and 60% indicating an adequate performance during the task.

The full factorial models including DHT or testosterone as a covariate of interest yielded a significant influence of both, DHT and testosterone, on individual neural reactivity, see Figure 2 and Table 2. Regarding the influence of DHT, a negative association was observed in left temporal regions for the contrast “stop error.” A positive relation was observed regarding testosterone in right temporal regions for the contrast “stop success.” There was also a positive relation observed for several temporal brain regions regarding the contrast stop success > stop error. No significant effect of medication was observed.

**FIGURE 2:**
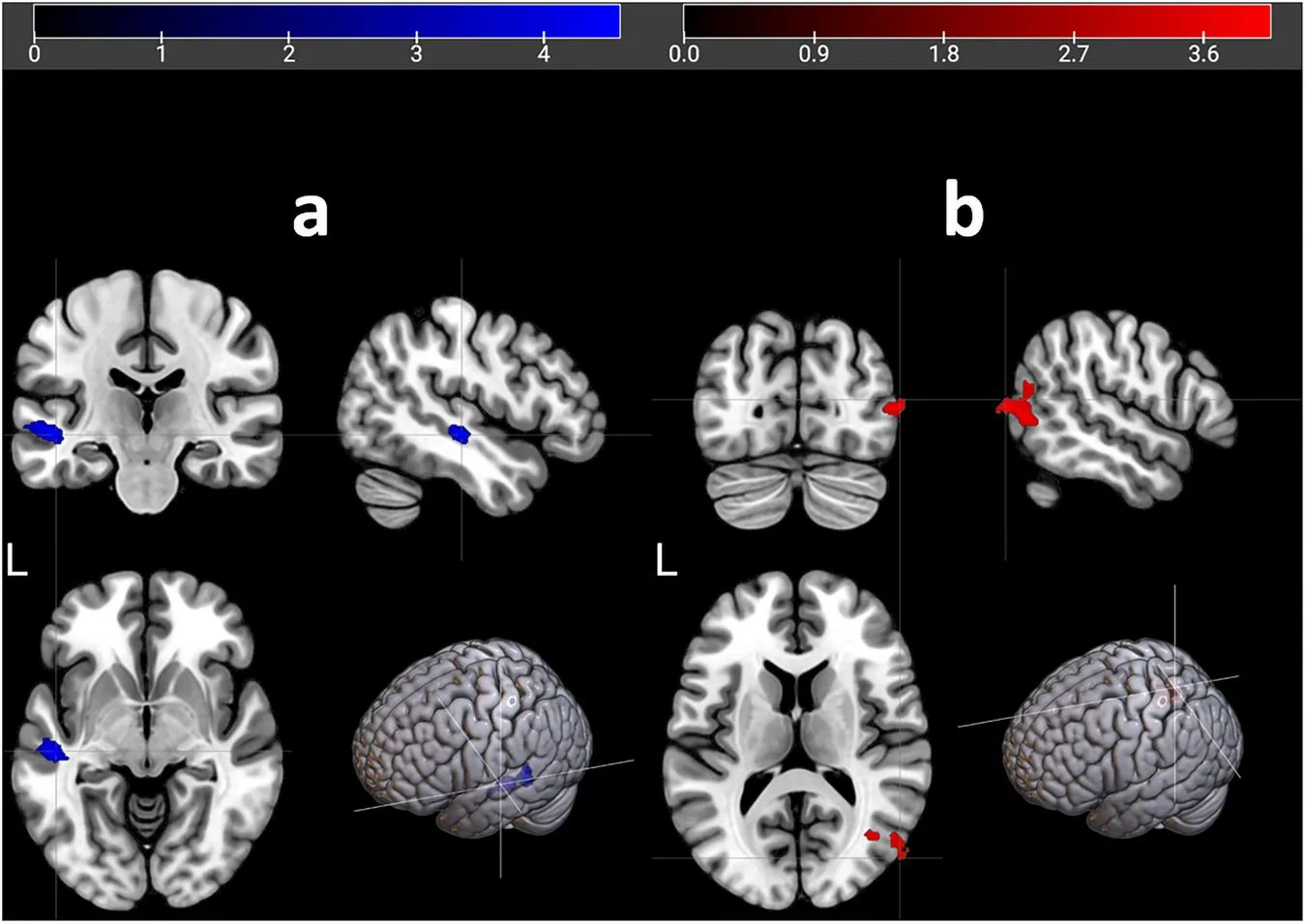
**(a)** Lower levels of DHT were associated with higher activation in left temporal regions for the contrast “stop error.” **(b)** Higher levels of T were associated with higher activation in right temporal regions for the contrast “stop success.” *N* = 45. An uncorrected threshold of *p* < 0.001 in combination with a cluster-extend threshold of panel **(a)** 353 voxels and **(b)** 321 voxels was used, corresponding to pFWE < 0.05. Coordinates: **(a)** xyz [–48, –22, –6], **(b)** xyz [54, –78, 12].

**TABLE 2:** Results from the full factorial fMRI analysis with factors subject, time point, sequence, and medication, including hormone levels as covariate of interest and age as covariate of no interest.

	Predictor	Peak MNI-coordinates			Brain area	Nr. of voxels *k*	Peak-level *t*(74)	Cluster-level *p*_*FWE–corrected*_
		x	y	z				
Stop error	Absolute DHT −	−48	−22	−6	Middle temporal gyrus L	353	4.75	0.005
	Sequence (placebo first < finasteride first)	−20	−74	14	Calcarine sulcus L, lingual gyrus L	927	4.41	<0.001
Stop success > stop error	Absolute DHT +	−48	−22	−6	Middle temporal gyrus L	282	4.65	0.016
		−2	−86	4	Calcarine sulcus L, lingual gyrus L, cuneus L	587	4.23	<0.001
		56	−20	4	Superior temporal gyrus	386	4.36	0.003
		−28	−32	38	Postcentral gyrus L	535	4.34	<0.001
Stop success	Absolute T +	54	−78	12	Middle temporal gyrus R	321	4.34	0.004
	Sequence (placebo first < finasteride first)	−22	−72	10	Calcarine sulcus L	215	4.39	0.030
		46	−4	24	Precentral gyrus R	277	4.10	0.009
		14	−6	54	Supplementary motor area R	192	4.38	0.047
	Time point (T1 > T2)	16	−10	6	Thalamus R	210	4.96	0.033

+Implicates positive prediction of brain activation; −Implicates negative prediction of brain activation. Only activated areas of significant voxel size in relation to total voxel size of a cluster are reported. Brain areas correspond to the Aal3 atlas [automated anatomic labeling atlas 3 (Rolls et al., 2020)]. L, left. R, right. No specification, both R and L. MNI-coordinates, Montreal Neurological Institute (Fonov et al., 2009). An uncorrected threshold of *p* < 0.001 in combination with a cluster-extend threshold of (a) 353 voxels and (b) 321 voxels was used, corresponding to pFWE < 0.05.

### 3.3 Behavioral results

The linear mixed models including with and without the factors time point, medication and sequence, as well as DHT or testosterone did not yield significant results with respect to behavioral measures (SSRT, go RT, SSD), except for an effect of age on the mean reaction times in go trials for both, DHT and testosterone. Please see Supplementary Figure 2 and Supplementary Table 4 for more details.

## 4 Discussion

Previous research demonstrated an association between impaired response inhibition and higher alcohol consumption, between impaired response inhibition and higher androgen levels, as well as between higher alcohol consumption and higher androgen levels. We demonstrated an association between lower DHT concentrations and higher activation in left temporal brain regions during a failure to inhibit. Additionally, we also observed that higher testosterone levels related to higher activation in right temporal regions during successful inhibitions. No significant findings were observed for behavioral variables.

In previous studies, patients with AUD showed increased activation in the middle temporal gyrus (Zeng et al., 2021) and decreased gray matter volume in this area compared to HCs (Yang et al., 2016). Right temporal regions, such as the right middle temporal gyrus, have been implicated in behavioral inhibition studies using the SST (Gan et al., 2014; Lenartowicz et al., 2011), and have further been found to be activated in inhibition studies using no-go trials (Vidal et al., 2012). The middle temporal gyrus has been connected to alcohol-induced impaired functioning in the SST (Gan et al., 2014). The left side of the middle temporal gyrus is not typically related to response inhibition. However, fetal androgen level (T) was found to predict gray matter volumes in the left middle temporal gyrus (Heany et al., 2016), making it seem likely that the activation of the left middle temporal gyrus in our study is driven by mechanisms related to testosterone and DHT. Following the observation by Moffat and Resnick (2007) in older males, higher free testosterone levels could be associated with increased cerebral blood flow in our sample.

Previous research indicates different regional responses for the contrasts “stop success” and “stop error” (Li et al., 2006) and distinct neural networks for stop success and stop error trials have been suggested (Zhang and Li, 2012). In agreement with our results, the right middle temporal gyrus forms a part of the network accounting for most variance in stop success trials as the right hemispheric network is more involved in attention to the stop signal, whereas the left hemispheric network is involved in response inhibition (Zhang and Li, 2012). Thus, our findings provide first evidence for an interaction of specific androgens with distinct neural processes during the SST, i.e., an influence of testosterone during attention to the stop signal (as supported more from the right hemisphere), and an influence of DHT during response inhibition (as supported more from the left hemisphere). In line with this argumentation, testosterone has been found to correlate positively with activation during spatial tasks and mental rotation in fMRI studies (Celec et al., 2015), tasks which also involve attention.

This study is the first to examine the role of DHT and the intertwining of androgens and inhibitory control in heavily drinking individuals. Thus, interpretations often need to infer from findings on effects of testosterone and further examination is warranted, e.g., using spectroscopy neuroimaging methods as well as confirmatory studies, as research on DHT mechanisms is still sparse. Hence, several aspects merit attention in this context: First, mechanisms underlying androgen effects, especially DHT and its reduction through finasteride, are still unclear, and as chronic alcohol consumption can lead to aromatization of androgens and testosterone is metabolized to estradiol (Purohit, 2000), it is possible that estradiol, which has also been suggested to play a role in problem drinking of males (Buchholz et al., 2019; Hoffmann et al., 2024), partly mediated the influence of testosterone in our study. Contrarily, DHT as the more potent derivative of testosterone therefore solely reflects an androgen-effect. Second, finasteride may not only exert its effects via the mechanism of 5-alpha reductase inhibition. Diviccaro et al. (2020) stated that finasteride does not only alter neuroactive steroid levels themselves but also their mechanisms of action. Indeed, finasteride acts on dopaminergic neurotransmitter systems, e.g., by blocking the effects of dopamine receptors in the mesolimbic system (Soggiu et al., 2016), and influences the inhibitory neurotransmitter system modulated by gamma-aminobutyric acid (Saengmearnuparp et al., 2021). Interestingly, previous research also suggested that finasteride interferes with functioning of the hypothalamus-pituitary-adrenal axis and thus impairs stress reactivity and reduces impulsive behavior (Godar et al., 2019). Third and while the specific mechanisms are still not clear, additional neural regions have previously been connected to behavioral inhibition, androgens and/or alcohol consumption. For instance, a significant association between higher testosterone levels and greater gray matter thickness in male adolescents were observed in the calcarine sulcus (Bramen et al., 2012), an area known to contain a high number of androgen receptors (Bramen et al., 2012), and sensitive to chronic effects of alcohol (Shokri-Kojori et al., 2017). The supplementary motor area (SMA) is frequently associated with inhibition, showing greater activation during successful inhibition in a group with shorter SSRTs (Li et al., 2006). In healthy adults, the preSMA is generally more active during successful stopping (Sharp et al., 2010). Hu et al. (2015) observed significantly greater activation in the preSMA in participants with AUD than in healthy controls during expectation of a stop signal - which they suggested to be a compensatory mechanism for cognitive control. Lastly, testosterone has been shown to influence uncertain social situations by acting on interpersonal trust via neuropeptide systems, thereby increasing social vigilance (Bos et al., 2012), thus indicating learning effects or neuroplasticity processes. Speculatively, finasteride could have triggered processes influencing social behavior in the unknown experimental situation. As vigilance is a key component of behavioral inhibition (Williams et al., 2020), the androgen effect of finasteride on social behavior might have influenced neural activation in the SST. Further, alcohol is used to facilitate social interaction (Müller et al., 2021, 2023) and modulation of DHT by alcohol consumption has been suggested as an underlying mechanism (Wenger et al., 2023). However, a previous study demonstrated that learning effects, in case of performing the SST multiple times, are not likely (You et al., 2023). Likewise, a pharmacological carry-over effect could be excluded, as the second examination day took place after at least 21 days; finasteride can remain functionally active after single administration for 1–2 weeks (Gisleskog et al., 1998).

## 5 Limitations

As previous research on the effect of 5-alpha reductase inhibition by finasteride on brain activation is very scarce, our findings are based on exploratory considerations. While in our sample a significant reduction of DHT after administration of finasteride was observed, the relative reduction was estimated at 9.6% on average, thus being smaller than the expected 80% (Gisleskog et al., 1998). This may be due to the single-dose administration of finasteride. Giechaskiel and Barouch (2024) state that the suppression of DHT is approximately 80% only in the long term, and DHT concentrations show greater variability following a single dose. Furthermore, the DHT reduction by 5 mg finasteride seems to be at its maximum a few hours after drug ingestion. DHT levels were reported to decrease to approximately 20% of baseline 2 h after drug ingestion. In our sample, the mean time between verum intake and serum sampling was 21.96 h (SD = 3.62). Accordingly, the effect of finasteride-induced DHT suppression may have been greater at an earlier time point. The resulting effect on behavioral and neural activity might thus have been too small to be detected in our sample (*N* = 45) and finasteride might have a generally smaller effect on neural activation during response inhibition in males with HED than expected.

Moreover, changes in neural activation do not necessarily translate into measurable behavioral differences, as it has been observed in previous, studies [e.g., (Honey and Bullmore, 2004; Liu et al., 2012; Vollstädt-Klein et al., 2019b)]. Subtle changes in the recruitment of task-related neural networks may be detectable with fMRI while remaining insufficient to affect behavioral performance.

Additionally, the test-retest reliability of the SST varies according to task contrast and brain region (Korucuoglu et al., 2021), which might interfere with possible small medication effects. Finally, we included only male subjects with HED. More research in larger samples is necessary to examine the effect of 5-alpha reductase inhibition by finasteride on brain activation in females and in other specific populations (i.e., AUD and HC), as well as additional mechanisms underlying the effect of finasteride on neural activity during response inhibition.

## 6 Clinical implications

The high prevalence of HED (Matos et al., 2016) and the fact that it is considered a major risk factor for AUD (Linden-Carmichael et al., 2017) underline the importance of developing novel preventive and therapeutic approaches. While a previous study reported positive effects of an inhibitor of 5-alpha reductase enzymes on abstinence and drinking outcomes in heavily drinking males (Covault et al., 2024), our study demonstrated a potential therapeutic approach as the pharmacological modulation of DHT influences neural correlates of response inhibition. However, the clinical and therapeutic potential of finasteride needs to examined further. Potential adverse effects should be considered when discussing the therapeutic relevance of 5α-reductase inhibition. In our sample, no adverse effects were reported and finasteride is generally well tolerated (Hirshburg et al., 2016). However, treatment has been associated with adverse effects, most commonly decreased libido, erectile dysfunction, and reduced ejaculate volume. Less frequent adverse effects include ejaculatory disorders, breast tenderness or enlargement, skin reactions, and mood-related symptoms such as depression or anxiety. Persistent sexual dysfunction has also been reported in a subset of patients after discontinuation, although the available evidence remains heterogeneous. Overall, adverse effects are considered uncommon and are often reversible after treatment cessation, but their potential occurrence should be carefully weighed in future studies evaluating finasteride as a possible intervention for alcohol-related disorders (Hirshburg et al., 2016).

## 7 Conclusion

We observed distinct associations between DHT and testosterone levels and activation patterns of temporal regions during a task measuring behavioral inhibition. These regions are involved in response inhibition, alcohol consumption, and androgen mechanisms, and results may point toward a role of DHT and testosterone in distinct hemispheric networks during the task. Although these findings should be interpreted with caution, they may inform future research aimed at further examining the role of androgens in alcohol-related behaviors. Further studies are needed to determine the pharmaceutical modulation of androgens with regards to their therapeutic potential for problem drinking or alcohol use disorder.

## Data Availability

The datasets presented in this article are not readily available due to ethical regulations. Requests to access the datasets should be directed to the corresponding author.
