# Supplementary material for: Pharmacologically altered androgens, brain activation and response inhibition in a stop-signal task in male heavy drinkers
Source: Front Syst Neurosci. 2026 Oct 1;20:1907416. doi: 10.3389/fnsys.2026.1907416 (PMC13669173; doi:10.3389/fnsys.2026.1907416)
Supplement: Supplementary file 1 [file Data_Sheet_1.pdf]

Supplement to

Pharmacologically altered Androgens, Brain Activation and Response Inhibition in a Stop-Signal Task in Male Heavy Drinkers

Marlene Kundlacz[1,2], Rafat Boroumand-Jazi[1], Sabine Hoffmann[1], Christiane Mühle[3], Iris Reinhard[4], Matthias Reichl[1], Leonard Wenger[1], Haoye Tan[1], Patrick Bach[1,5], Sabine Vollstädt-Klein[1,5,7], Georg W. Alpers[2], Falk Kiefer[1,5,6,7], Bernd Lenz[1,5,7*#], Sarah Gerhardt[1,5#]

[1]Department of Addictive Behavior and Addiction Medicine, Central Institute of Mental Health (CIMH), Medical Faculty Mannheim, Heidelberg University, Mannheim, Germany.

[2]Otto Selz Institute and Department of Psychology, School of Social Sciences, University of Mannheim, Mannheim, Germany.

[3]Department of Psychiatry and Psychotherapy, Friedrich-Alexander-Universität Erlangen-Nürnberg (FAU), Erlangen, Germany.

[4]Core Facility Biostatistics, Central Institute of Mental Health (CIMH), Medical Faculty Mannheim, Heidelberg University, Mannheim, Germany

[5]Deutsches Zentrum für Psychische Gesundheit (German Center for Mental Health), Partner Site Mannheim-Heidelberg-Ulm.

[6]Feuerlein Center on Translational Addiction Medicine (FCTS), Heidelberg University, Germany.

[7]Mannheim Centre for Translational Neurosciences (MCTN), Medical Faculty Mannheim, Heidelberg University, Mannheim, Germany

*Corresponding author at: Department of Addictive Behavior and Addiction Medicine, Central Institute of Mental Health (CIMH), Medical Faculty Mannheim, Heidelberg University, J 5, 68159 Mannheim, Germany. Phone: +49 621 1703-3522, Fax: +49 621 1703-3505. E-Mail: bernd.lenz@zi-mannheim.de

[#]both authors contributed equally

1. Inclusion and exclusion criteria

Inclusion criteria

- Male sex
- Age 18 – 65 years
- Body Mass Index 18.5 – 30 kg/m^2
- Heavy episodic drinking (five or more drinks on one occasion at least once a month)
- Ability to consent after detailed written information and ability to undergo functional magnetic resonance imaging measurement
- Written informed consent
- Sufficiently good German
- Breath alcohol concentration of 0.0 per mille

Exclusion criteria

- Medical history of outpatient or inpatient treatment of depressive or bipolar disorder, schizophrenia or schizophrenic spectrum disorder, or substance use disorder other than alcohol, nicotine and cannabis
- Medical history of a severe head injury or other serious diseases of the central nervous system (e.g. dementia, Parkinson's disease, multiple sclerosis)
- Intake of any drugs or medication that interact with the central nervous system
- Intake of any drugs or medication that interact with the central nervous system within the last 10 days or less than 5 half-lives ago
- Exclusion criteria for use of finasteride: regular use of finasteride, known hypersensitivity to finasteride, clinically significant pharmacokinetic interactions, planned pregnancy with sexual intercourse without contraception or pregnant partner
- Exclusion criteria for functional magnetic resonance imaging measurement (e.g. metal in the body

2. Figure S1 displays available data and dropouts according to the CONSORT flow diagram

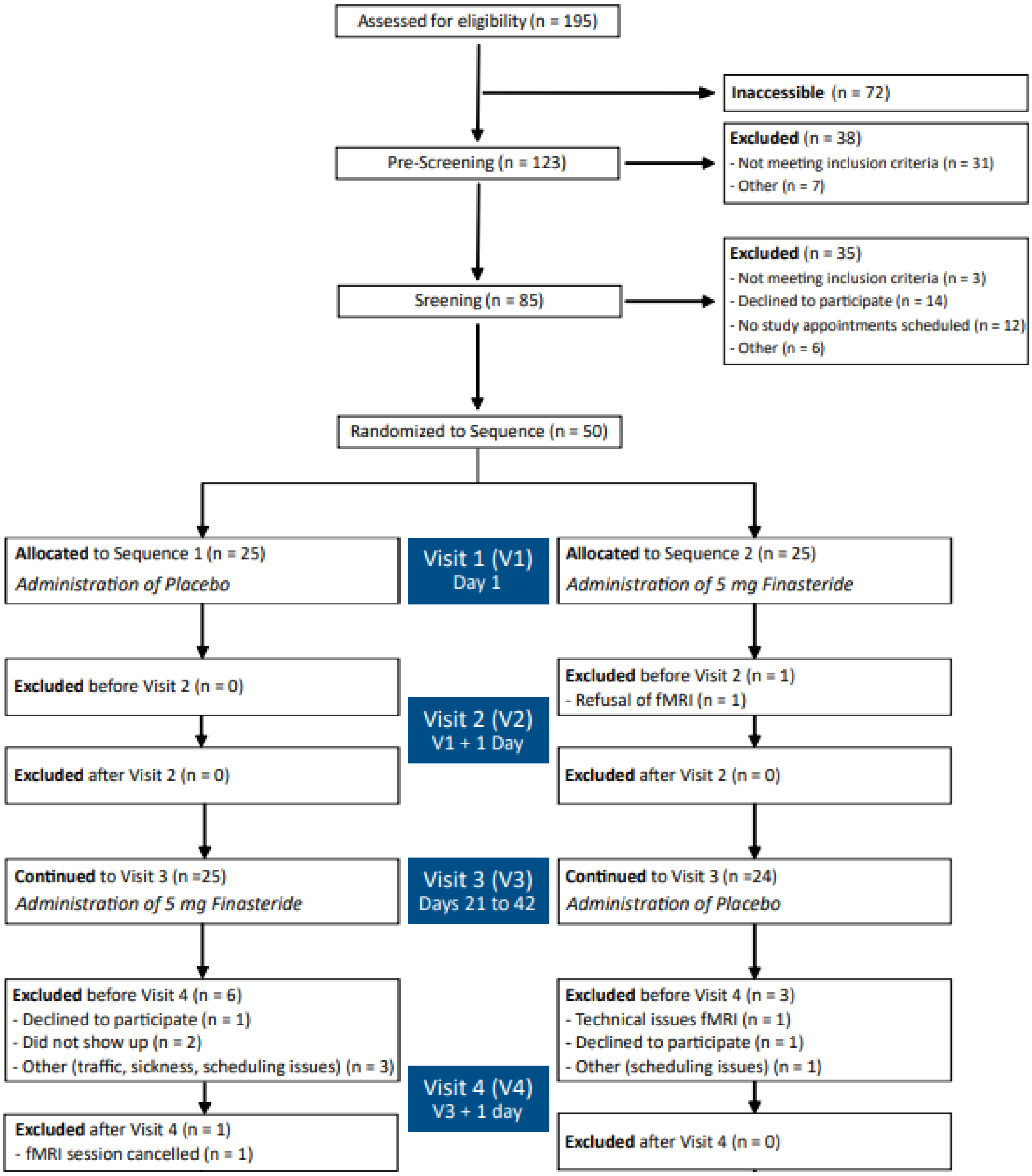

Note: Study visits V2 and V4 represent the first and the second fMRI measurement (T1 and T2), respectively. Overall, N = 47 participants presented analyzable data sets with N = 32 participants having two analyzable fMRI data sets, and N = 15 only one.
For the sequence Finasteride-Placebo, N = 3 participants were excluded from the analysis for the first, and N = 4 for the second assessment time point. For the sequence Placebo-Finasteride, N = 2 participants were excluded from the analysis for the first, and N = 0 for the second assessment time point. This was due to heavy movement in the scanner (N = 5) and technical problems (N = 4).
This results in N = 21 analyzable data sets for the first, and N = 17 analyzable date sets for the second assessment time point for the sequence Finasteride-Placebo, and N = 23 and N = 18 for the sequence Placebo-Finasteride, respectively.

3. Contrasts as specified for the full factorial model in SPM12

Table S1: Titles of contrasts and numbers as entered for weights vectors in the full factorial model with hormones as an active covariate

Factor	Name of Contrast	Weights vector
Time point	Main time T1 > T2	1 -1 0 0 0.5 0.5 -0.5 -0.5 0
Time point	Main time T1 < T2	-1 1 0 0 -0.5 -0.5 0.5 0.5 0
	Interaction Time point x Sequence	0 0 0 0 1 -1 -1 1 0
Sequence	Main sequence Placebo-Medication > Medication-Placebo	0 0 1 -1 0.5 -0.5 0.5 -0.5 0
Sequence	Main sequence Placebo-Medication < Medication - Placebo	0 0 -1 1 -0.5 0.5 -0.5 0.5 0
Covariate	Influence hormones (+)	0 0 0 0 0 0 0 0 1
Covariate	Influence hormones (-)	0 0 0 0 0 0 0 0 -1

Note: Main, Main Effect. *Interaction*, Interaction effect. T1 and T2 correspond to V2 and V4, respectively. The nine weight vectors (regressors) correspond to main effects of T1 (1) and T2 (2), sequence 1 (3) and sequence 2 (4), and the hormone covariate (9). Vectors 5 to 8 result from the applied full factorial model and represent factor weights as well.

4. Sample characteristics at baseline (visit one)

Table S2: Sample characteristics at baseline by sequence

Sequence	Fin - Pla		Pla - Fin		Mean difference
	M	*SD*	*M*	*SD*	
Age	33.95	14.40	33.04	13.03	$t(45) = .23$, $p = .82$
Height (cm)	182.50	7.60	182.72	7.14	$t(45) = -.10$, $p = .92$
Weight (kg)	84.05	13.84	81.8	9.75	$t(45) = .65$, $p = .52$
AUDIT	10.77	5.11	11.83	4.97	$t(45) = -.71$, $p = .48$
BIS (sum score)	30.73	5.70	29.52	5.11	$t(45) = .77$, $p = .45$

*Note: S*tatistics for mean differences were conducted by using independent samples t-Tests in SPSS (IBM SPSS Statistics, Version 29.0, 2022). *Fin,* Finasteride. *Pla,* Placebo. *M,* mean. *SD,* standard deviation. *AUDIT,* Alcohol Use Disorders Identification Test. *BIS,* Baratt Impulsiveness Scale.

5. Results tables for whole brain analyses

Table S3: Significantly activated brain regions during 'stop success', 'stop error', and 'stop success vs. stop error' on the whole brain level for placebo only (*n* = 38) in a one sample t-Test in SPM12

Contrast	Nr. of voxels per cluster *k*	Brain regions	Peak MNI-Coordinates x y z	Extent threshold	Peak-Level *t*	Cluster-Level $p_{FWE\ corrected}$
ss > se	614	Cluster 1 ss<se: Frontal inferior opercular gyrus L, Insula L	-48 8 4	212	4.48	<.001
	634	Cluster 2 ss<se: Postcentral gyrus L, Parietal inferior gyrus L	-58 -22 50	212	6.41	<.001
	354	Cluster 3 ss<se: Precentral gyrus R, Postcentral gyrus R	54 -16 50	212	4.70	.004
	324	Cluster 4 ss>se: Paracentral lobule, Supplementary Motor Area R	12 -22 58	324	4.85	.007
ss	13821	Cluster 1 ss>0: Temporal middle gyrus R, Occipital middle gyrus R, Supramarginal gyrus R, Temporal superior gyrus R	50 -66 0	258	9.76	<.001
	10015	Cluster 2 ss>0: Occipital middle gyrus L, Temporal middle gyrus L, Fusiform L	-48 -66 6	258	11.07	<.001
	8817	Cluster 3 ss>0: Frontal inferior opercular gyrus R, Precentral gyrus, Insula R, Supplementary motor area	34 20 4	258	8.71	<.001
	1828	Cluster 4 ss>0: Insula L	-32 20 -8	258	8.80	<.001
	621	Cluster 5 ss<0: Precuneus	-16 -52 18	225	4.97	<.001
	412	Cluster 6 ss>0: Frontal middle gyrus R	28 48 18	258	4.55	.007
	391	Cluster 7 ss<0: Angular gyrus L	-42 -72 32	225	5.28	.001
	225	Cluster 8 ss<0: Postcentral gyrus L	-56 -16 50	225	4.27	0.014
	804	Cluster 9 ss<0: Precentral gyrus R, Postcentral gyrus R	34 -28 72	225	5.33	<.001

se	534	Cluster 1 se<0: Cerebelum R	40 -68 -40	534	5.50	<.001
	8444	Cluster 2 se>0: Occipital middle gyrus L, Parietal inferior gyrus L, Temporal middle gyrus L, Fusiform L	-40 -56 -14	318	12.30	<.001
	11169	Cluster 3 se>0: Temporal middle gyrus R, Supramarginal gyrus R	48 -66 0	318	10.70	<.001
	1016	Cluster 4 se<0: Frontal medial orbital gyrus, Rectus, Frontal inferior orbital gyrus L	4 44 -18	534	5.97	<.001
	3873	Cluster 5 se>0: Insula L, Precentral gyrus L, Frontal inferior opercular gyrus L	-32 22 10	318	10.77	<.001
	9691	Cluster 6 se>0: Frontal inferior opercular gyrus R, Supplementary motor area	40 16 2	318	9.94	<.001
	954	Cluster 7 se<0: Precuneus	36 -44 0	534	5.24	<.001
	885	Cluster 8 se<0: Angular gyrus L, Occipital middle gyrus L	-44 -76 38	534	5.97	<.001
	1014	Cluster 9 se<0: Frontal superior gyrus L, Frontal middle gyrus L	-24 20 38	534	5.41	<.001

Note: Regions are labelled according to the automated anatomical labelling atlas 3 (Rolls, Huang, Lin, Feng, & Joliot, 2020). The analyses were corrected combing a voxel-wise-threshold of $p_{uncorr} < 0.001$ with a cluster-extend-threshold determined with random field theory in SPM12 for $p_{FWE} < 0.05$. *MNI-Coordinates*, Montreal Neurological Institute (Fonov, Evans, McKinstry, Almli, & Collins, 2009). *Ss,* stop success. *Se,* stop error. *R,* right. *L,* left. No specification, both R and L

A successful implementation of the SST paradigm in our sample was demonstrated by significant neural activation in regions relevant for behavioral control using fMRI data from the placebo condition (e.g., (Bednarski et al., 2012; Li, Luo, Yan, Bergquist, & Sinha, 2009; Ray Li, Huang, Constable, & Sinha, 2006)).

6. The stop-signal task (SST)

The well-established setup of the SST is designed for participants to reach an average of 50% stop successes and 50% stop errors (see (Gan et al., 2014)) for more details). To reach approximately 50% stop successes and stop errors (i.e., probability of inhibition (PI)), the time that passed between a simple signal (arrow pointing to the right or left) and a stop signal (arrow pointing upwards) was dynamically adapted throughout the experiment. This so-called stop signal delay time (SSD) was increased by 50 ms following a successfully inhibited response and decreased by 50 ms in case of a failure to inhibit. Accordingly, a longer SSD indicated better inhibitory performance, as inhibition was still successful although the stop signal appeared with more delay to the signal before. Additionally, reaction times (RT) and the percentage of successful stop trials were assessed, and the stop signal reaction time (SSRT) was calculated to estimate response latency of response inhibition according to Gan and colleagues (Gan et al., 2014). Overall, good test-retest reliability is known regarding neural correlates of the SST for the inferior and middle frontal, superior parietal, and precentral gyri for correct stop vs. correct go trials (i.e., successful inhibition) (Korucuoglu et al., 2021).

7. Mixed model analyses with behavioral data

Table S4: Results from mixed models analyses with behavioral data from the SST as outcome variables

	β [95% CI]	*F*(df1, df2)	*p*
**DHT only**			
**Mean SSRT (msec)**			
Intercept	262.28 [163.78; 360.78]	*F*(1, 75) = 28.13	**<.001**
DHT post	-.13 [-.32; .06]	*F*(1, 76) = 1.83	.180
Age	.78 [-.88; 2.45]	*F*(1, 46) = .89	.349
**Mean go RT (msec)**			
Intercept	388.21 [248.17; 528.26]	*F*(1, 77) = 30.47	**<.001**
DHT post	.13 [-.13; .39]	*F*(1, 72) = 1.03	.313
Age	3.12 [.63; 5.61]	*F*(1, 48) = 6.36	**.015**
**Mean SSD**			
Intercept	96.02 [-127.88; 319.93]	*F*(1, 77) = .73	.396
DHT post	.35 [-.07; .78]	*F*(1, 77) = 2.70	.104
Age	2.47 [-1.35; 6.28]	*F*(1, 48) = 1.69	.200
**DHT with factors**			
**Mean SSRT (msec)**			
Intercept	285.85 [178.50; 393.20]	*F*(1, 72) = 26.54	**<.001**
Time point	-3.03 [-29.71; 23.64]	*F*(1, 34) = 0.52	.475

medication	-6.69 [-45.98; 32.61]	$F(1, 40) = 0.12$	.733
Sequence	-20.08 [-68.88; 28.73]	$F(1, 42) = 1.07$	.308
DHT post	-.15 [-.37; .06]	$F(1, 76) = 2.11$	.150
Age	.73 [-.95; 2.42]	$F(1, 46) = 0.77$	.386
**Mean go RT (msec)**			
Intercept	355.91 [203.55; 508.27]	$F(1, 74) = 23.25$	**<.001**
Time point	-21.58 [-55.87; 12.70]	$F(1, 36) = 0.66$	.421
medication	24.79 [-25.97; 75.54]	$F(1, 41) = 0.97$	.330
Sequence	21.87 [-50.31; 94.06]	$F(1, 44) = 1.00$	.322
DHT post	.21 [-.08; .50]	$F(1, 72) = 2.03$	.159
Age	3.16 [.64; 5.68]	$F(1, 47) = 6.36$	**.015**
**Mean SSD (msec)**			
Intercept	58.95 [-184.12; 302.02]	$F(1, 73) = 0.48$	.492
Time point	-20.04 [-80.09; 40.01]	$F(1, 37) = 0.58$	.450
medication	9.98 [-77.93; 97.89]	$F(1, 43) = 0.05$	.820
Sequence	62.82 [-47.59; 173.24]	$F(1, 44) = 1.73$	.195
DHT post	.40 [-.07; .88]	$F(1, 77) = 2.84$	.096
Age	2.36 [1.47; 6.18]	$F(1, 47) = 1.53$	.222
**Testosterone only**			
**Mean SSRT (msec)**			
Intercept	248.83 [149.39; 348.26]	$F(1, 70) = 24.92$	**<:001**
T post	-6.65 [-19.91; 6.60]	$F(1, 79) = 1.00$	.321
Age	.83 [-.84; 2.51]	$F(1, 44) = 1.00$	.322
**Mean go RT (msec)**			
Intercept	355.99 [214.79; 497.18]	$F(1, 75) = 25.23$	**<.001**
T post	14.44 [-3.68; 32.56]	$F(1, 76) = 2.52$	.117
Age	3.37 [.86; 5.89]	$F(1, 48) = 7.26$	**.010**
**Mean SSD (msec)**			
Intercept	119.08 [-107.84; 346.01]	$F(1, 72) = 1.09$	.299
T post	20.53 [-9.52; 50.57]	$F(1, 80) = 1.85$	.178
Age	2.42 [-1.44; 6.28]	$F(1, 48) = 1.59$	.213
**Testosterone with factors**			
**Mean SSRT (msec)**			
Intercept	286.92 [175.82; 398.01]	$F(1, 69) = 27.37$	**<.001**
Time point	-11.05 [-37.44; 15.35]	$F(1, 33) = 0.75$	.394
medication	6.24 [-30.83; 43.30]	$F(1, 33) = 0.12$	.734
Sequence	-30.65 [-80.33; 19.03]	$F(1, 42) = 1.46$	.234
T post	-9.73 [-23.60; 4.14]	$F(1, 76) = 1.95$	.166
Age	.71 [-.96; 2.38]	$F(1, 44) = 0.74$	.393
**Mean go RT (msec)**			
Intercept	334.29 [177.36; 491.21]	$F(1, 73) = 23.30$	**<.001**
Time point	-9.95 [-42.42; 22.52]	$F(1, 36) = 0.33$	.571
medication	6.95 [-38.61; 52.50]	$F(1, 36) = 0.10$	.759

Sequence	39.21 [-34.48; 112.90]	*F*(1, 46) = 1.51	.225
T post	16.14 [-2.80; 35.09]	*F*(1, 73) = 2.88	.094
Age	3.30 [.78; 5.82]	*F*(1, 48) = 6.95	**.011**
**Mean SSD (msec)**			
Intercept	53.90 [-197.72; 305.51]	*F*(1, 71) = 0.66	.420
Time point	1.22 [-57.18; 59.63]	*F*(1, 37) = 0.30	.586
medication	-24.65 [-105.73; 56.44]	*F*(1, 37) = 0.38	.542
Sequence	91.63 [-21.29; 204.56]	*F*(1, 45) = 2.30	.137
T post	25.75 [-5.49; 56.99]	*F*(1, 77) = 2.69	.105
Age	2.41 [-1.41; 6.24]	*F*(1, 47) = 1.62	.210

Note. *95% CI*, 95% confidence interval. *β,* regression coefficient. *Msec*, milli seconds. *SSRT*, stop signal reaction time. *RT*, reaction time. *SSD*, stop signal delay. Age and DHT (post medication/placebo administration) were included as covariates. The multilevel models include fixed and random intercepts. Significant results ($p < 0.05$) are highlighted in bold.

8. Figure S2: Scatterplots displaying raw behavioral data from the SST in relation to DHT and testosterone. Top row: DHT. Bottom row: testosterone. From left to right panels: mean stop signal reaction time, mean stop signal delay, mean go reaction time. Blue lines and dots indicate hormone serum levels following placebo medication, green lines and dots indicate hormone serum levels following finasteride intake.

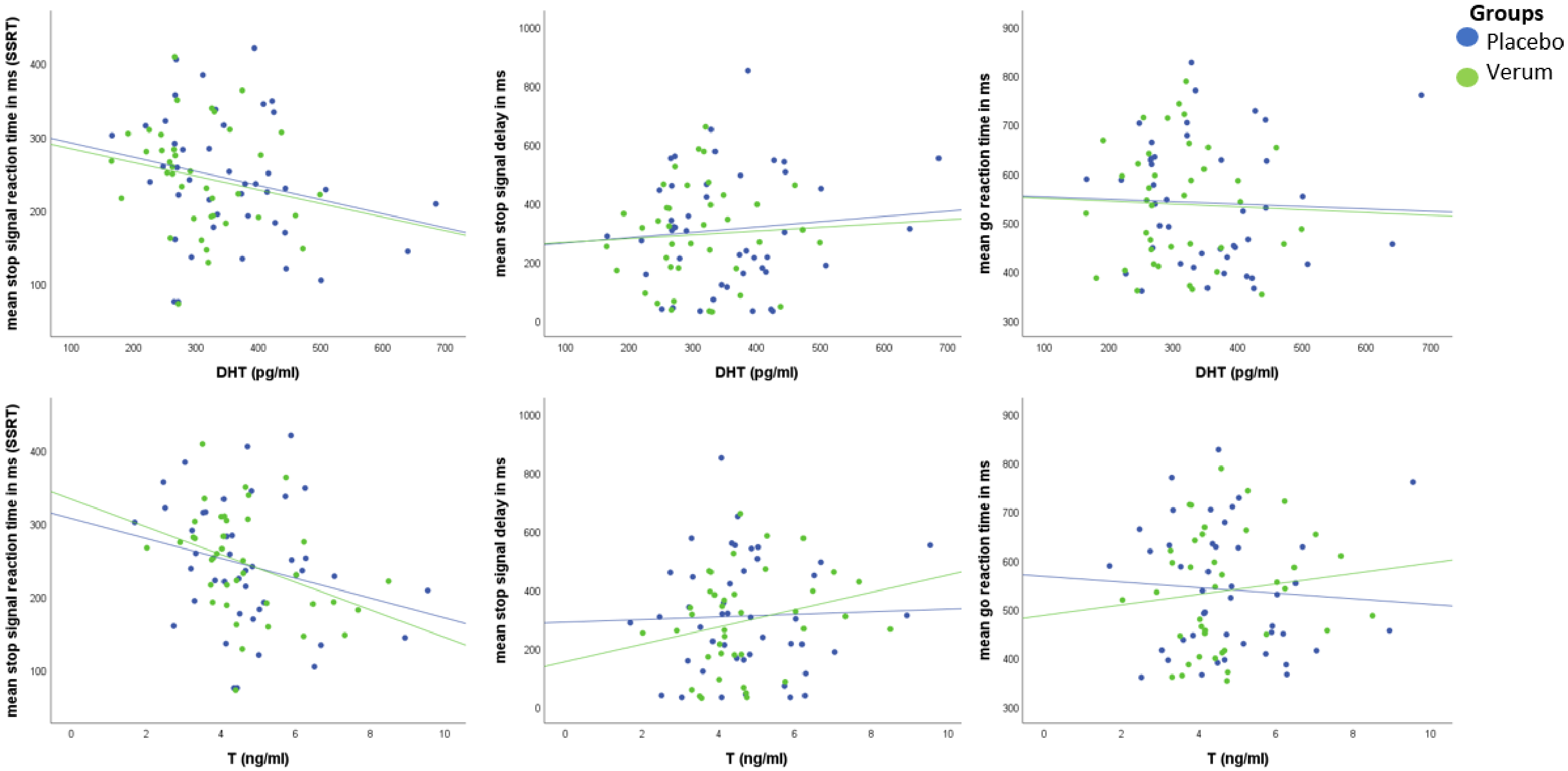

References

Bednarski, S. R., Erdman, E., Luo, X., Zhang, S., Hu, S., & Li, C. S. R. (2012). Neural Processes of an Indirect Analog of Risk Taking in Young Nondependent Adult Alcohol Drinkers—An fMRI Study of the Stop Signal Task. *Alcoholism: Clinical and Experimental Research, 36*(5), 768-779. doi:10.1111/j.1530-0277.2011.01672.x

Faul, F., Erdfelder, E., Lang, A.-G., & Buchner, A. (2007). G*Power 3: A flexible statistical power analysis program for the social, behavioral, and biomedical sciences. *Behavior Research Methods, 39*(2), 175-191. doi:10.3758/bf03193146

Ferguson, C. J. (2016). *An effect size primer: A guide for clinicians and researchers*. Washington, DC, US: American Psychological Association.

Fonov, V. S., Evans, A. C., McKinstry, R. C., Almli, C. R., & Collins, D. L. (2009). Unbiased nonlinear average age-appropriate brain templates from birth to adulthood. *NeuroImage, 47*, S102. doi:https://doi.org/10.1016/S1053-8119(09)70884-5

Gan, G., Guevara, A., Marxen, M., Neumann, M., Jünger, E., Kobiella, A., . . . Smolka, M. N. (2014). Alcohol-Induced Impairment of Inhibitory Control Is Linked to Attenuated Brain Responses in Right Fronto-Temporal Cortex. *Biological Psychiatry, 76*(9), 698-707. doi:https://doi.org/10.1016/j.biopsych.2013.12.017

Korucuoglu, O., Harms, M. P., Astafiev, S. V., Golosheykin, S., Kennedy, J. T., Barch, D. M., & Anokhin, A. P. (2021). Test-Retest Reliability of Neural Correlates of Response Inhibition and Error Monitoring: An fMRI Study of a Stop-Signal Task. *Frontiers in Neuroscience, 15*. doi:10.3389/fnins.2021.624911

Li, C. S. R., Luo, X., Yan, P., Bergquist, K., & Sinha, R. (2009). Altered Impulse Control in Alcohol Dependence: Neural Measures of Stop Signal Performance. *Alcoholism: Clinical and Experimental Research, 33*(4), 740-750. doi:10.1111/j.1530-0277.2008.00891.x

Ray Li, C.-S., Huang, C., Constable, R. T., & Sinha, R. (2006). Imaging Response Inhibition in a Stop-Signal Task: Neural Correlates Independent of Signal Monitoring and Post-Response Processing. *The Journal of Neuroscience, 26*(1), 186-192. doi:10.1523/jneurosci.3741-05.2006

Rolls, E. T., Huang, C.-C., Lin, C.-P., Feng, J., & Joliot, M. (2020). Automated anatomical labelling atlas 3. *NeuroImage, 206*, 116189. doi:https://doi.org/10.1016/j.neuroimage.2019.116189
